# GmMYB183, a R2R3-MYB Transcription Factor in Tamba Black Soybean (*Glycine max*. cv. Tamba), Conferred Aluminum Tolerance in *Arabidopsis* and Soybean

**DOI:** 10.3390/biom14060724

**Published:** 2024-06-19

**Authors:** Yunmin Wei, Rongrong Han, Yongxiong Yu

**Affiliations:** 1 College of Life Sciences and Oceanography, Shenzhen University, Shenzhen 518060, China; weiym1024@szu.edu.cn; 2 College of Optoelectronic Engineering, Shenzhen University, Shenzhen 518060, China; 3 College of Animal Science and Technology, Southwest University, Chongqing 400715, China; 0062926@swu.edu.cn; 4 Chongqing College of Traditional Chinese Medicine, Chongqing 402760, China

**Keywords:** GmMYB183, transcription factor, citrate secretion, Al tolerance, phosphorylation, abiotic stress

## Abstract

Aluminum (Al) toxicity is one of the environmental stress factors that affects crop growth, development, and productivity. MYB transcription factors play crucial roles in responding to biotic or abiotic stresses. However, the roles of MYB transcription factors in Al tolerance have not been clearly elucidated. Here, we found that *GmMYB183*, a gene encoding a R2R3 MYB transcription factor, is involved in Al tolerance. Subcellular localization studies revealed that GmMYB183 protein is located in the nucleus, cytoplasm and cell membrane. Overexpression of *GmMYB183* in *Arabidopsis* and soybean hairy roots enhanced plant tolerance towards Al stress compared to the wild type, with higher citrate secretion and less Al accumulation. Furthermore, we showed that GmMYB183 binds the *GmMATE75* gene promoter encoding for a plasma-membrane-localized citrate transporter. Through a dual-luciferase reporter system and yeast one hybrid, the GmMYB183 protein was shown to directly activate the transcription of *GmMATE75*. Furthermore, the expression of *GmMATE75* may depend on phosphorylation of Ser36 residues in GmMYB183 and two MYB sites in P3 segment of the *GmMATE75* promoter. In conclusion, GmMYB183 conferred Al tolerance by promoting the secretion of citrate, which provides a scientific basis for further elucidating the mechanism of plant Al resistance.

## 1. Introduction

Aluminum (Al), the most abundant metallic element in the Earth’s crust, is solubilized into a toxic trivalent cation (Al^3+^) in acidic soils with a pH value of less than 5.0. This solubilization inhibits plant root growth, blocking nutrients and water intake, resulting in severe losses in crop production [1,2]. Approximately 50% of the world’s potential arable land is acidic, and soil acidification is increasing due to industrial pollution and modern farming practices [3,4]. Therefore, Al toxicity presents a huge threat to agricultural production and productivity. 

Plants employ a variety of strategies to mitigate the harmful effects of Al stress. One such strategy is the secretion of organic acids induced by Al, which plays a crucial role in most plant species [5,6,7]. Several plant species, including barley (*Hordeum vulgare*) [8], cabbage (*Brassica oleracea*) [9], camelina (*Camelina sativa*) [10], rape (*Brassica napus*) [11,12], rubber tree (*Hevea brasiliensis*) [13], and wheat (*Triticum aestivum*) [14], have been found to secrete malate, which forms chelates with Al and detoxifies it, whereas eucalyptus (*Eucalyptus camaldulensis*) [15], maize (*Zea mays*) [16], populous (*Populous trichocarpa*) [17], rice bean (*Vigna umbellata*) [18,19,20], *Psychotria* (*Psychotria rubra*) [21], sorghum (*Sorghum bicolor*) [22], soybean (*Glycine max*) [23,24,25], and wild soybean (*Glycine soja*) [26] have been shown to secrete citrate, which aids in the detoxification of Al. Additionally, the upregulation of transporters responsible for the secretion of citrate or malate enhances a plant’s tolerance to Al [1,6].

Transcription factors such as zinc finger proteins, WRKY, HD-ZIP, or NAC are known to play crucial roles in Al tolerance. One example is STOP1/ART1, a zinc finger transcription factor, which regulates the expression of various genes related to Al tolerance in different crop species, such as Arabidopsis (*Arabidopsis thaliana*) [27], cotton (*Gossypium hirsutum*) [28], pigeonpea (*Cajanus caja*) [29], rice (*Oryza sativa*) [30], sorghum, soybean [31] or tobacco (*Nicotiana tabacum*) [32]. Although the transcription of STOP1 is unaffected by Al stress, its activities at the post-transcriptional or post-translational levels are regulated by Al stress [33,34]. The SUMOylation of STOP1 has been found to be involved in modulating Al tolerance, suggesting that post-translational modifications of transcription factors are crucial in Al stress responses [35]. Similarly, WRKY22 has been shown to enhance Al tolerance by increasing the expression of OsFRDL4 and promoting citrate secretion in rice [36]. Another transcription factor, HvHOX9, a member of the HD-ZIP family, mediates Al resistance in barley roots by inhibiting Al binding to the root cell wall and increasing the apoplastic pH [37]. Furthermore, VuNAR1, a NAC transcription factor, promotes Al tolerance by activating WAK1 expression and regulating cell wall pectin metabolism [38]. However, the exact roles of the MYB family of genes in Al tolerance have not been fully elucidated.

In plants, the MYB family of transcription factors is considered one of the largest families and plays significant roles in regulating gene transcription networks involved in various developmental and stress-response mechanisms [39]. A study conducted by Wei et al. demonstrated that the overexpression of *TaMYB344* in tobacco confers tolerance to heat, drought, and salt-induced stress [40]. Similarly, Shin et al. found that StMYB1R-1 activates genes associated with drought tolerance, leading to improved drought resistance in potatoes [41]. Additionally, the gene ARS1, which encodes the R1-MYB type transcription factor, is induced by salinity and contributes to salt tolerance in tomato leaves [42]. Similarly, GsMYB7 has been identified as a potential regulator of soybean’s tolerance to acidic aluminum stress through the regulation of downstream genes [43]. Hence, MYB transcription factors are believed to play crucial roles in plants’ response to Al stress.

Tamba black soybean (TBS) is an Al-tolerant genotype with significant potential for Al tolerance via the secretion of citrate under Al stress (Figure S1). Previously, we studied the effect of GmMYB183 on the Al stress response in TBS. We observed significant phosphorylation shifts, including upregulated phosphorylation of a serine residue (Ser36) [44]. In this study, our objective was to further investigate the function of GmMYB183 in the Al stress response. Our findings revealed that overexpression of *GmMYB183* leads to increased tolerance to Al in transgenic *Arabidopsis* and soybean hairy roots. Additionally, we discovered that GmMYB183 directly binds to the promoter of *GmMATE75*, resulting in enhanced expression of this gene. In addition, GmMYB183 that binds to the MYB sites in the P3 segment of the *GmMATE75* promoter may depend on phosphorylation of Ser36 residues in GmMYB183.

## 2. Materials and Methods

### 2.1. Plant Culture and Treatment

TBS seedlings were cultured as previously described [44]. Briefly, pre-treated roots were transferred to a 50 µmol/L AlCl_3_ solution (containing 0.5 mmol/L CaCl_2_, pH 4.5) for 3 h, 6 h, 9 h, 12 h, 24 h, 48 h or 96 h. Arabidopsis seeds (ecotype Columbia, Col-0) were directly germinated on mixed soil (substrate: vermiculite = 1:1) and grown in a controlled environment at day/night temperatures of 25/22 °C, with 16 h of light (120 µmol photons m^−2^·s^−1^).

### 2.2. GmMYB183 Gene Isolation and Sequence Analysis

Based on the previous analysis of the quantitative phosphoproteomics of TBS, we observed that, under acidic aluminum exposure, GmMYB183 was hyperphosphorylated at Ser36. We then obtained the *GmMYB183* gene sequence data from Phytozome 12 database, using a gene ID, Glyma.06G187600. The specific primers of GmMYB183 amplification were designed from the full-length cDNA, which were used to clone the gene from TBS roots using RT-PCR. The primers in this study are shown in Table S1.

We used the ExPaSy platform (https://www.expasy.org/, accessed on 24 February 2020) to predict physical and chemical properties of the protein. Localization of the GmMYB183 gene was predicted using NetNES 1.1, and then the BLAST tool was used to search for GmMYB183 homologous proteins in the NCBI database (https://blast.ncbi.nlm.nih.gov/Blast.cgi, accessed on 24 February 2020). Conserved domains and the three-dimensional structure of the GmMYB183 protein were predicted using the Conserved Domain Search (https://www.ncbi.nlm.nih.gov/Structure/cdd/wrpsb.cgi, accessed on 24 February 2020) and PDB software (http://www.sbg.bio.ic.ac.uk/phyre2/html/page.cgi?id=index, accessed on 24 February 2020). In addition, DNAMAN was used to perform homology studies of the GmMYB183 protein. Then, the MEGA 7.0 software was used to construct phylogenetic trees through the neighbor-joining method with 1000 bootstrap replications.

### 2.3. RNA Extraction and Quantitative Real-Time PCR (qRT-qPCR)

Total RNA was extracted using the RNAi^so^ Plus kit (Takara, Shiga, Japan) according to the manufacturer’s instructions. First-strand cDNA was synthesized using Prime Script^TM^ reagent Kit with a gDNA Eraser kit (Takara, Shiga, Japan). Quantitative real-time PCR (RT-qPCR) was performed according to our previous study with *40SrRNA* gene (GenBank: XM_003549836.4) as an internal control [44]. Primers used in this study are presented in Table S1.

### 2.4. Vector Construction and Transformation of GmMYB183 into Arabidopsis and Soybean Hairy Roots

Encoding region of *GmMYB183* cDNA was ligated into a pMD™ 19-T vector (Takara, Shiga, Japan). Fragments encoding *GmMYB183* were amplified from the pMD™ 19-T-*GmMYB183* vector using a pair of specific primers with terminal *Xba* I and *Sma* I restriction sites (Table S1). Moreover, the coding region of the *GmMYB183* gene was mutated using Mut Express^®^ II Fast Mutagenesis Kit V2 (Vazyme, Nanjing, China) according to the manufacturer’s instructions. Expression vectors, pBI121-*GmMYB183*-*eGFP* (OE) and pBI121-*GmMYB183-S36A*-*eGFP* (OE-m), were created by inserting the coding region of the *GmMYB183* gene into the same restriction sites under the control of the CaMV 35S promoter and NOS terminator of the expression box. Then, pBI121-*eGFP*, pBI121-*GmMYB183*-*eGFP* and pBI121-*GmMYB183-S36A*-*eGFP* were introduced into the *Agrobacterium tumefaciens* strain GV3101 through the heat shock method. Full-flowering Arabidopsis plants with fruit pods and flowers were used for genetic transformation through the floral dip method. The surface of Arabidopsis seeds was disinfected with 7% sodium hypochlorite for 10 min, washed with sterile water for three times, then spread on the medium of 1/2 MS (containing 500 mg/L Cefixime). After germination, the Arabidopsis seedlings with green fluorescence were screened out using an LUYOR-3415RG hand-held fluorescent protein excitation light source (as indicated by the arrow in Figure S2). The expression of *GmMYB183* was further detected by RT-qPCR. The seeds of T0 generation Arabidopsis were disinfected and cultured on 1/2 MS medium. The single-copy T1 generation of transgenic Arabidopsis thaliana was screened according to the ratio of transgenic/wild type = 3:1. Among them, three single-copy lines of OE-1, OE-3 and OE-7 were screened in *GmMYB183* transgenic lines, and three single-copy lines of OE-m2, OE-m5 and OE-m6 were screened in *GmMYB183-S36A* transgenic lines. Homozygous T2 and T3 generations were further screened for subsequent studies.

The full-length coding sequence of GmMYB183, amplified from the pMD™ 19-T-*GmMYB183* vector, was sub-cloned into the pBin35S-*Red3* vector between *Eco*R I and *Xho* I restriction sites, downstream of the 35S promoter to generate a *GmMYB183* overexpression vector (OX). The empty pBin35S-*Red3* vector was used as the negative control (EV). Site-directed mutations were established as above. The *GmMYB183* overexpressing vector and the empty pBin35S-*Red3* vector were introduced into the *Agrobacterium rhizogenes* strain K599 for transformation of black soybean hairy roots. Soybeans germinated in the soil for 4–5 days, and samples were taken before cotyledon was fully developed. The hypocotyls with cotyledon were cut 1 cm away from cotyledon and used as explants. The cotyledon was infected in the prepared infection solution for 1 h, and the positive hair roots were observed and screened after 14 days of moisturized culture.

### 2.5. Subcellular Localization of the GmMYB183 Protein

For subcellular localization, Arabidopsis roots were mounted on glass slides, covered and viewed using an inverted LSM800 laser scanning microscope (Carl Zeiss, Oberkochen Germany). The empty pBI121-*eGFP* vector was used as the negative control.

### 2.6. Phenotypic Identification of GmMYB183 Transgenic Lines

The Arabidopsis seeds were surface sterilized with 7% sodium hypochlorite for 15 min, washed three times in sterile water, and then incubated in 1/2 MS agar medium. For relative root growth, the seedlings, after germination for 3 d, were transplanted onto a 0, 50, 100, and 150 μmol/L AlCl_3_ solution (containing 0.5 mmol/L CaCl_2_, pH 4.5) for 7 d. The root relative growth was evaluated according to the procedures described by Min et al. [45]. We then determined citrate secretion and hematoxylin staining according to our previous work [46]. All treatments were performed in triplicates, and each replicate contained 3 plants.

### 2.7. Yeast Two-Hybrid and Yeast One-Hybrid Assays

To evaluate the interaction between the GmMYB183 and 14-3-3 protein (GmSGFa), yeast two-hybrid assays were carried out using AH109 yeast strain, according to the manufacturer’s instructions. The *GmSGFa* gene was cloned into the pGADT7 vector to generate the pGADT7-*GmSGFa* construct, while the *GmMYB183* or *GmMYB183-S36A* was cloned into pGBKT7 vector to generate the pGBKT7-*GmMYB183* or pGBKT7-*GmMYB183-S36A* construct. The AH109 yeast strain was co-transformed with different construct combinations and incubated in a medium lacking Trp (T), Leu (L), His (H), and Adenine (A), but with 10 mmol/L 3-AT.

On the other hand, to evaluate the binding of GmMYB183 to the *GmMATE75* promoter, yeast one-hybrid assays were performed according to the procedures described by Yu et al. [47]. Briefly, the full-length GmMYB183 gene was ligated between the *EcoR*I and *Xho*I sites and fused in frame in yeast GAL4-AD effector plasmids. The four fragments of the *GmMATE75* promoter were inserted into p178 vector to generate reporter plasmids. Subsequently, the different plasmid combinations were co-transformed into EGY48 yeast strain, and the interactions were tested on an SD medium lacking Trp and Ura, for 2~3 days, at 28 °C. Positive clones were incubated on X-gal plates with filter paper for 8 h at 28 °C.

### 2.8. Dual-LUC Assays

Dual-LUC assays were performed to quantify the binding affinity of the GmMYB183 protein to the *GmMATE75* gene promoter. The ORFs of *GmMYB183* were ligated into *Xba* I and *Sma* I restriction sites of pGreenII 62-SK vector to generate 35S-GmMYB183 effector plasmids. On the other hand, the *GmMATE75* promoter was cloned into pGreen 0800-Luc at the *Xho* I and *Hind* III restriction sites to generate proGmMATE75-Luc reporter plasmids. The 35S-GmMYB183 vector was mutated by PCR to generate 35S-GmMYB183-S36A, while proGmMATE75-Luc vector was mutated by PCR to generate proGmMATE75-m1-Luc, proGmMATE75-m2-Luc and proGmMATE75-m1m2-Luc. The reporter and effector vectors were transformed into *Agrobacterium tumefaciens* strain GV3101 (pSoup). We then conducted infiltration in tobacco leaves and detection following the described protocols [48].

### 2.9. Statistical Analysis

Data for the relative expression, root growth, citrate secretion, and LUC/REN were presented as a mean ± the standard error of the mean (SEM). The data were processed using SPSS Statistics19 using Duncan’s test. We used GraphPad (Version 8.3.0) for graph preparation and presentation. A *p* < 0.05 was considered to be statistically significant.

## 3. Results

### 3.1. In Silico Analysis of the GmMYB183 Gene

Based on data from quantitative phosphoproteomics of TBS, a protein with significantly up-regulated phosphorylation induced by Al stress was screened [44]. The full-length coding sequences (CDSs) of *GmMYB183* were amplified using cDNA from TBS. The sequencing results showed that the CDS of GmMYB183 is 885 bp, encoding 294 amino acids, with a MW of 31.87 kDa. The gene sequence was consistent with the sequence of soybean genomic MYB183 (NM_001249070.1). Furthermore, NetNES 1.1 analysis showed that the nuclear export signal was located at the C-terminal of 257–263 amino acids (Figure S3a), and CDD analysis revealed that a SANT conserved the domain of 77–126 amino acids in the GmMYB183 protein, with an E value of 1.30 × 10^−14^ (Figure S3b). Prediction of the GmMYB183 secondary structure by SOPMA showed that α-helix accounted for 21.77%, extension chain accounted for 20.75%, β-corner accounted for 6.46%, and irregular crimping accounted for 51.02% (Figure S3c). In addition, the three-dimensional (3d) structure was successfully established by PDB software (Figure S3d). 

**Figure 1:**
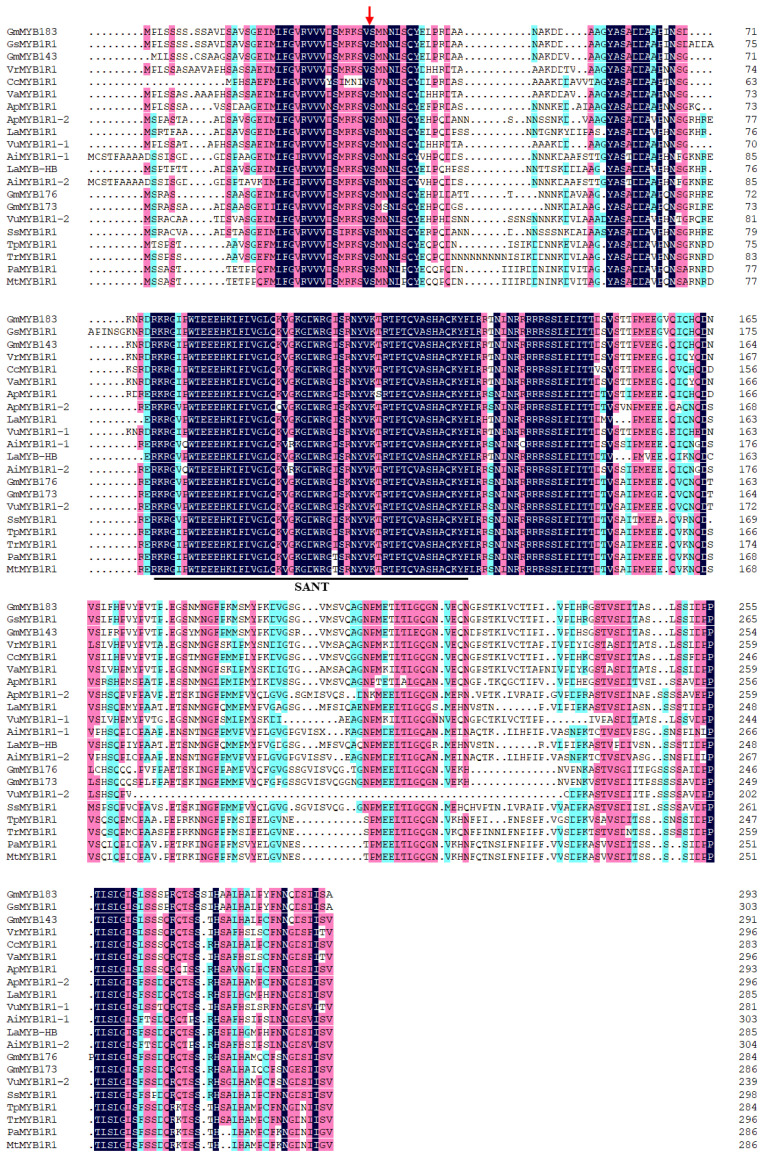
Homologous alignment analysis of GmMYB183. Red arrow represents phosphorylation sites and the underscore presents the SANT domain. The accession number of each protein is listed in Table S2.

Homology analysis of GmMYB183 amino acid sequences revealed a typical SANT MYB domain, with high similarities in the N-terminal DNA-binding domain, as well as in the C-terminal sequence (Figure 1). In addition, evolutionary relatedness, as evaluated by MEGA7.0, revealed the highest homology (92.83%) between the GmMYB183 of TBS and soybean GmMYB143, and were clustered into one branch with MYB transcription factors of other legumes (Figure S4).

### 3.2. Expression Profiles of GmMYB183 in Tissues and Pertubations by Aluminum Stress

**Figure 2:**
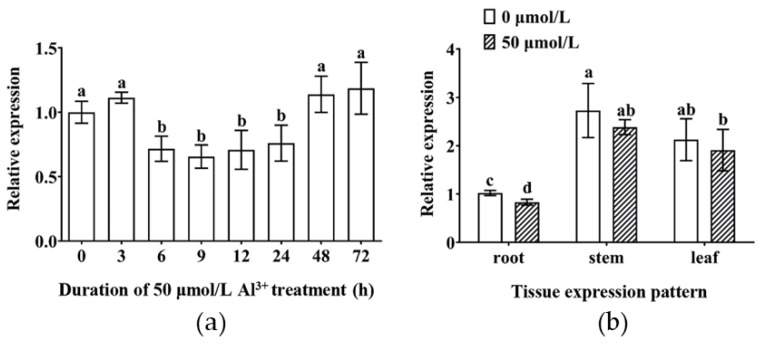
Expression patterns of *GmMYB183* in tissues and under Al^3+^ treatment. (**a**) Temporal expression pattern of *GmMYB183* in root tips under Al^3+^ treatment. Seedlings were exposed to 50 µmol/L AlCl_3_, and the 2 cm long roots were taken from the seedlings at the processing time nodes of 0, 3, 6, 9, 12, 24, 48 and 72 h. (**b**) Tissue expression patterns of *GmMYB183*. Samples of roots, stems, and leaves are from seedlings treated with 0 or 50 µmol/L AlCl_3_ for 24 h. Column bars represent means ± standard errors (n = 3). Different letters represent significant differences (Duncan, *p* < 0.05).

To evaluate the expression shifts of the TBS *GmMYB183* gene under Al stress, we performed qRT-PCR analysis. Expression levels of *GmMYB183* were found to be significantly suppressed within 6–24 h treatment of 50 µmol/L Al^3+^ in root tips, suggesting that *GmMYB183* may be involved in the Al stress response (Figure 2a). Furthermore, expression levels of *GmMYB183* in stems and leaves were found to be elevated when compared to those in the roots (Figure 2b). However, there were no significant differences in the expression levels in roots, stems and leaves compared with the control after Al stress for 24 h (Figure 2b).

### 3.3. Subcellular Localization of the GmMYB183 Protein 

**Figure 3:**
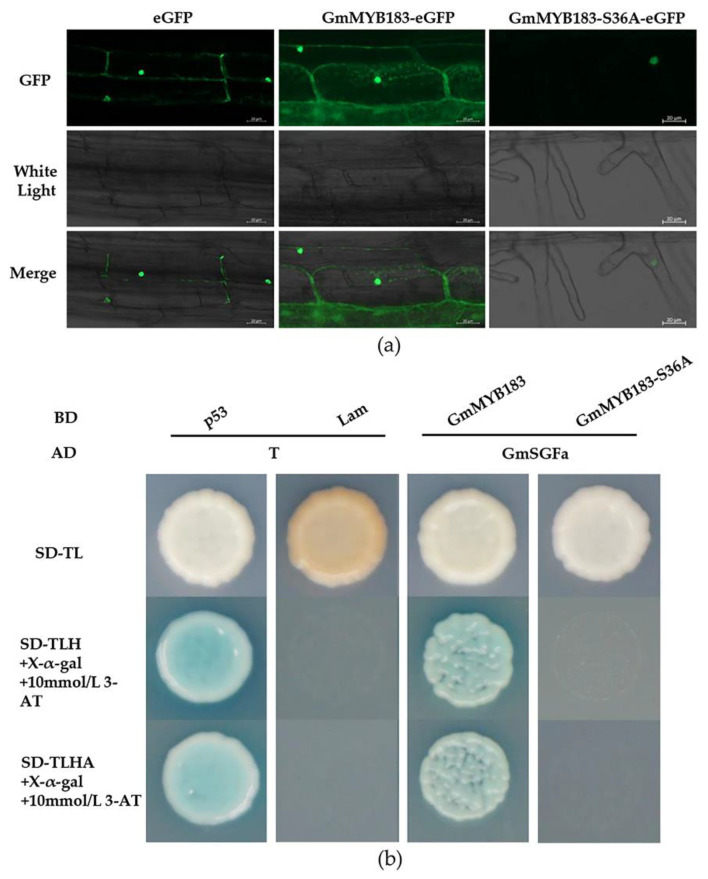
Subcellular localization analysis of the GmMYB183 protein in Arabidopsis root cells. (**a**) Subcellular localization of the GmMYB183 and GmMYB183-S36A protein. (**b**) Evaluation of how GmMYB183 or GmMYB183-S36A interacts with GmSGFa using yeast two hybrid.

To evaluate the localization of the GmMYB183 protein, a full-length *GmMYB183* gene was fused to the N-terminal of the eGFP (enhanced green fluorescent protein) reporter gene under a CaMV35S promoter. The eGFP fluorescence signals were analyzed in Arabidopsis root cells via Agrobacterium-mediated transformation. It was found that 35S-eGFP control and GmMYB183-eGFP were localized in both the nucleus and cytoplasmic membrane (Figure 3a). Furthermore, for the mutated Ser36 GmMYB183-S36A-eGFP, the signal was predominantly in the nucleus (Figure 3a). 

Previous studies have shown that 14-3-3 proteins regulate the subcellular localization of phosphorylated proteins [49]. In addition, the Al stimuli enhanced the interaction between 14-3-3 protein and phosphorylated plasma membrane H^+^-ATPase, thereby promoting citrate secretion [50,51]. Furthermore, our previous data showed that Al stress significantly increased the expression of the *14-3-3a* gene (*GmSGFa*) in TBS roots. We speculated that, through interaction with GmSGFa, GmMYB183 might change its subcellular localization. The interactions of GmMYB183 with GmMYB183-S36A and GmSGFa were studied using the yeast two-hybrid system, and as expected, the GmMYB183 protein was shown to interact with GmSGFa, but not GmMYB183-S36A (Figure 3b).

### 3.4. Phenotypic Identification of GmMYB183 Transgenic Arabidopsis 

**Figure 4:**
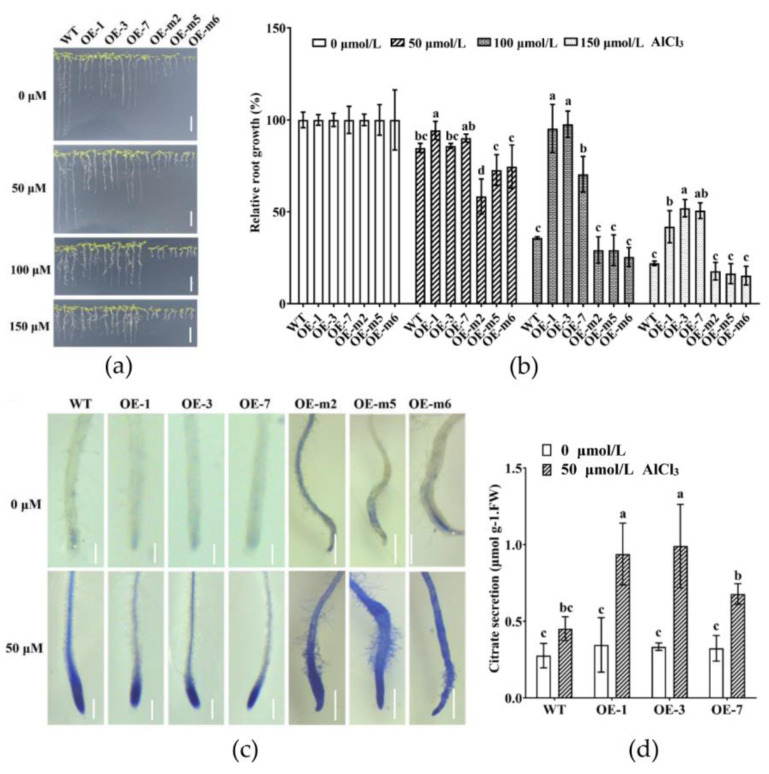
Overexpression of the *GmMYB183* gene improved Al tolerance in Arabidopsis. (**a**) After germination in 1/2 MS medium, three-day-old seedlings were transferred to solid medium containing 0, 50, 100, and 150 μmol/L AlCl_3_ (500 μmol/L CaCl_2_, pH 4.5) for 7 days (scale bar = 1 cm). (**b**) Relative root growth after treatment with various AlCl_3_ concentrations for 7 days. (**c**) Hematoxylin staining for roots treated with 50 μmol/L AlCl_3_ (scale bar = 1 mm). (**d**) Citrate secretion of roots treated with various AlCl_3_ concentrations for 24 h. OE: Overexpression of *GmMYB183* in Arabidopsis; OE-m: Overexpression of *GmMYB183-S36A* in Arabidopsis. Columns and bars represent means ± standard errors (n = 3). Different letters represent significant differences (Duncan, *p* < 0.05).

According to the above results, we speculate that GmMYB183 plays an important role in the regulation of the tolerance to Al stress. To evaluate the role of GmMYB183 in Al tolerance, six homozygous T3 transgenic Arabidopsis lines were carefully chosen for subsequent phenotypic and physiological analysis (Figure S5). Relative root growth is one of the most important indices for evaluating Al tolerance in plants [52]. Exposure to Al stress exerted a concentration-dependent inhibition of Arabidopsis root growth (Figure 4a). The root growth of transgenic Arabidopsis plants was diminished. In addition, under Al stress, the root growth of transgenic plants overexpressing *GmMYB183-S36A* were shorter than those of WT plants. However, unlike WT plants, transgenic plants overexpressing *GmMYB183* exhibited relatively longer root growth under Al stress (Figure 4b). Moreover, compared to WT, upon hematoxylin staining, transgenic plant roots overexpressing *GmMYB183* or *GmMYB183-S36A* showed lighter or deeper staining, respectively (Figure 4c). In addition, citrate secretions in transgenic plants overexpressing *GmMYB183* were significantly higher compared to those of WT (Figure 4d), consistent with the expression of the citrate transporter gene (Figure S6). These findings imply that overexpression of *GmMYB183* enhanced citrate secretion to alleviate Al toxicity in Arabidopsis.

### 3.5. Overexpression of GmMYB183 in Soybean Hairy Roots Confers Al Tolerance

**Figure 5:**
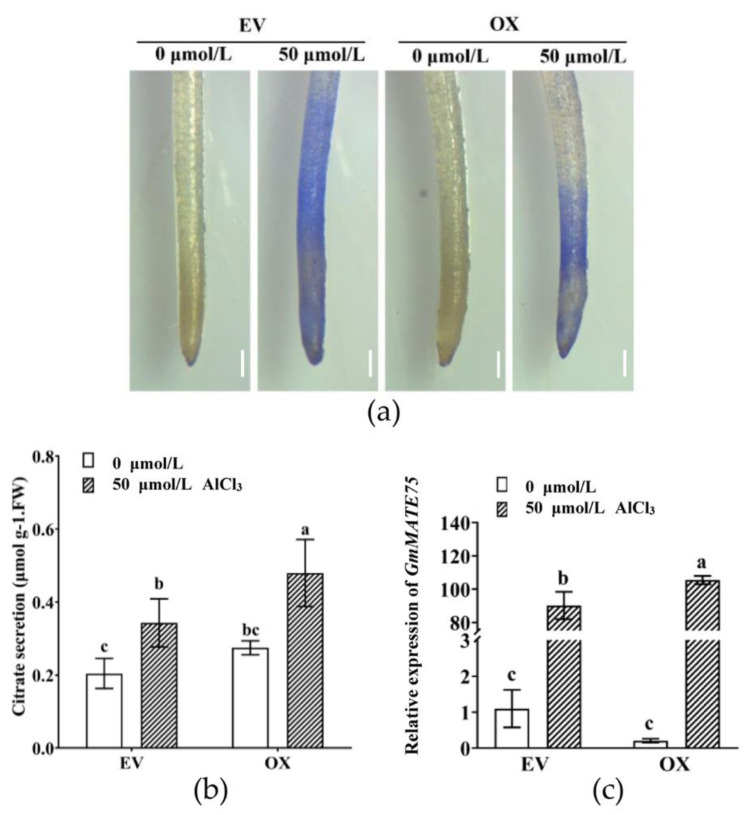
Overexpression of the *GmMYB183* gene improves Al tolerance in soybean hairy roots. (**a**) Hematoxylin staining of hairy roots after 50 μmol/L AlCl_3_ treatment (scale bar = 1 mm). (**b**) Citrate secretion of hairy roots treated with various AlCl_3_ concentrations for 24 h. (**c**) Relative expression of *GmMATE75* in hairy roots. EV: empty vector; OX: overexpression of *GmMYB183* in soybean hairy root. Bars on columns represent means ± standard errors (n = 3). Different letters represent significant differences (Duncan, *p* < 0.05).

To further evaluate the role of *GmMYB183* in Al stress responses, 35S::Red and 35S::GmMYB183::Red constructs were introduced into soybean hairy roots (Figures S7 and S8) [53]. The hematoxylin staining degree of the OX root tip was lighter than that of EV (Figure 5a). Furthermore, citrate secretion from the OX root tip was found to be significantly higher than those of EV (Figure 5b), consistent with findings from Arabidopsis. In addition, under Al stress, expression levels of Al-tolerance genes such as *GmMATE75* in OX were significantly higher than that in EV (Figure 5c), implying that GmMYB183 enhanced citrate secretion by promoting *GmMATE75* expression.

### 3.6. GmMYB183 Binds to the GmMATE75 Promoter 

**Figure 6:**
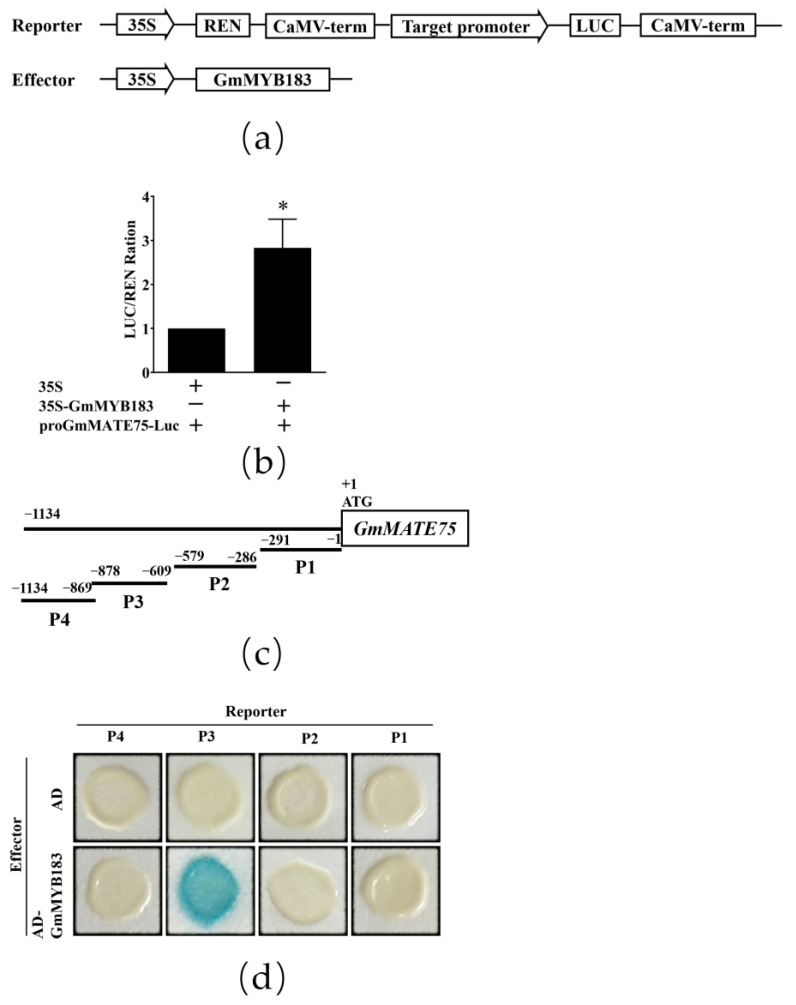
Dual-luciferase and yeast one-hybrid analysis of GmMYB183 and promoters of the *GmMATE75* gene. (**a**) Vector construction of transient expression assay. (**b**) Dual-luciferase assays between GmMYB183 and promoters of the *GmMATE75* gene. (**c**) The *GmMATE75* promoter region was divided into four fragments (P1, P2, P3 and P4). (**d**) Yeast one-hybrid assays. LUC, Firefly luciferase activity; REN, Renilla luciferase activity (control). Bars on columns represent means ± standard errors (n = 3). * above columns represent a significant difference (Duncan, *p* < 0.05).

*GmMATE75*, a gene encoding the citrate transporter, has been shown to enhance Al tolerance through Al-induced citrate efflux [23]. Using the 5′ rapid amplification of the cDNA ends (RACE) technique, we identified a transcriptional initiation site of the *GmMATE75* gene, and analyzed MYB binding sites in the *GmMATE75* gene promoter using PlantCare software (http://bioinformatics.psb.ugent.be/webtools/plantcare/html/, accessed on 19 April 2019) (Figure S9). Interactions between GmMYB183 and the *GmMATE*75 gene promoter were tested using the double luciferase detection system (Figure 6a). It was found that GmMYB183 exhibited much higher activity of the luciferase reporter gene compared to the empty vector control (Figure 6b). The yeast one-hybrid (Y1H) assay was also used to determine whether GmMYB183 binds MYB regions of the *GmMATE75* promoter (Figure 6c). However, the yeast strain, EGY48, co-transformed with pB42AD-GmMYB183 and P3-reporter was the only one that showed the blue color (Figure 6d). Therefore, GmMYB183 positively regulates *GmMATE75* by binding the P3 segment of the *GmMATE75* promoter.

**Figure 7:**
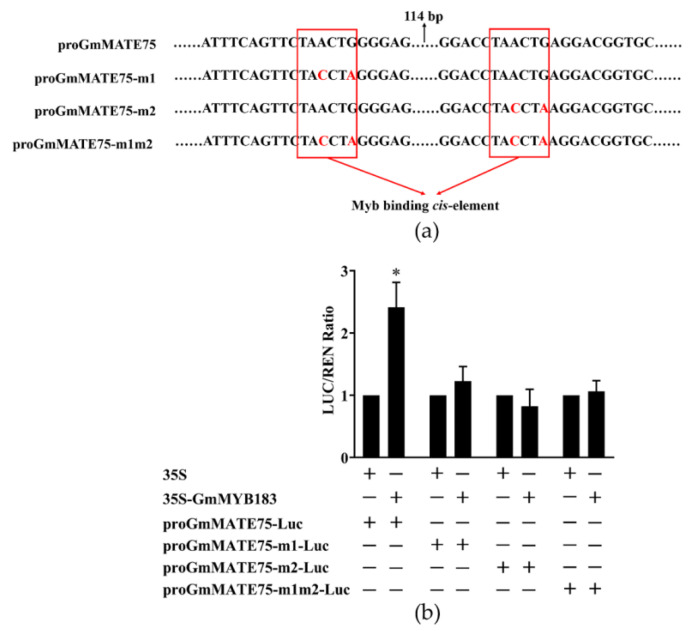
Dual-luciferase analysis of GmMYB183 and promoters of the *GmMATE75* gene, without or with mutation sites. (**a**) Sequences of *GmMATE75* gene promoters without or with mutation site. (**b**) Dual-luciferase assays between GmMYB183 and P3 segments of the *GmMATE75* promoter. LUC, Firefly luciferase activity; REN, Renilla luciferase activity. Columns and bars represent means ± standard errors (n = 3). * above columns represent a significant difference (Duncan, *p* < 0.05).

To further analyze the transcriptional regulation of *GmMATE75* by GmMYB183, we designed mutation primers Mut-proGmMATE75-1 or Mut-proGmMATE75-2 to mutate two MYB sites in the P3 segment of the *GmMATE75* promoter, and generated promoter-Luc reporter constructs (pGreenII 0800-proGmMATE75-m1-Luc, pGreenII 0800-proGmMATE75-m2-Luc and pGreenII 0800-proGmMATE75-m1m2-Luc) (Figure 7a). Double luciferase activity detection revealed that GmMYB183 activates the expression of GmMATE75, and the expression is a function of the two MYB sites (Figure 7b).

### 3.7. GmMYB183 Regulates GmMATE75-Dependent S36 Phosphorylation

**Figure 8:**
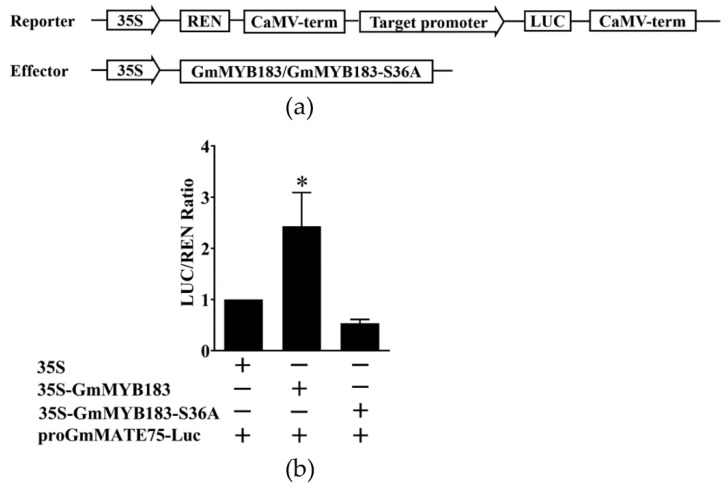
Dual-luciferase analysis of GmMYB183, GmMYB183-S36A and promoters of the *GmMATE75* gene. (**a**) Dual-luciferase assays between GmMYB183 and promoters of the *GmMATE75* gene. (**b**) Analysis of double luciferase activity. LUC, Firefly luciferase activity; REN, Renilla luciferase activity. Bars on columns represent means ± standard errors (n = 3). * above columns represent a significant difference (Duncan, *p* < 0.05).

To investigate the importance of Ser36 phosphorylation in the GmMYB183-specific regulation of *GmMATE75*, Ser36 was mutated to alanine after which the activity of the luciferase gene was analyzed using the double luciferase detection system (Figure 8a). Whereas GmMYB183 promoted the activity of the *GmMATE75* promoter, the mutated GmMYB183-S36A did not (Figure 8b). These data show that GmMYB183 activity may depend on the phosphorylation of its Ser36 in regulating the expression of *GmMATE75*.

## 4. Discussion

Al toxicity is a crucial factor that significantly restrains plant growth and crop productivity in acidic soils [1]. Recent studies have shed light on the regulation of Al-tolerance-related genes by transcription factors [54,55,56,57,58]. Nonetheless, research exploring the post-translational modification of transcription factors in response to Al stress is scarce. Remarkable shifts in phosphorylation have been observed in GmMYB183 in response to Al stress, without any alterations in the transcript levels of GmMYB183 [44]. Interestingly, the phosphorylation of ALR1, a recently discovered Al receptor, is also regulated by Al stress [59]. With increasing Al concentration, the phosphorylation of ALR1-BAK1 increased, similar to GmMYB183, which may partially explain the concentration-dependent release of organic acids from roots under Al stress. In addition, Al stress induces the kinase activity of MPK4, which interacts with and phosphorylates STOP1 [60]. Hence, it is more probable that GmMYB183 contributes to Al tolerance through their phosphorylation activities. However, the mechanism by which ALR1 regulates GmMYB183 remains unclear. Further studies are necessary to provide a scientific basis for elucidating the mechanism of plant Al tolerance.

Hematoxylin staining has been employed to assess the levels of stress tolerance in soybean. For instance, it was observed that roots overexpressing the *GmME1* or *GsGIS3* genes displayed minimal hematoxylin staining compared to WT, following exposure to Al stress, indicating a robust resistance to Al [61,62]. In this study, faint staining of the *GmMYB183-OE* root tips in both Arabidopsis and soybean hairy roots was consistent with the relative growth of roots in Arabidopsis plants (Figure 4). Regarding further conditions, transgenic GmMYB183 plants exhibited significantly higher levels of citrate secretion compared to the WT under Al stress (Figure 5b). These findings demonstrate that overexpression of *GmMYB183* enhances citrate secretion, thereby conferring Al tolerance in Arabidopsis and soybean hairy roots. In addition, citrate secretion could potentially alter the apoplastic pH of the root environment, impacting the availability of nutrients and the activity of enzymes involved in root growth. It is worth mentioning that citrate secretion can improve the utilization capacity of soil phosphorus and reduce the use of phosphorus fertilizer in acidic soil. Therefore, the role of GmMYB183 under abiotic stress is also worthy of our investigation. MATE transporters play multiple roles in plants including detoxification, secondary metabolite transport, Al tolerance, and disease resistance. To investigate the potential molecular regulatory mechanisms of GmMYB183 under Al stress, we profiled several Al-tolerance-related genes in WT or transgenic lines. RT-qPCR analysis revealed that, under Al stress, *GmMATE75* was upregulated in *GmMYB183-OX* root tips, which might be responsible for improved Al tolerance in transgenic plants (Figure 5c). GmMATE75, a plasma-membrane-localized citrate transporter, mediates Al-induced citrate efflux in soybean root apices [23]. Our previous plasma membrane proteomics analysis showed that, under Al stress, GmMATE75 is upregulated in Al-tolerant TBS [63]. In this study, we found that GmMYB183 binds the promoter of soybean *GmMATE75* genes. Similarly, Li et al. reported that OsWRKY22 activates *OsFRDL4* expression and enhances citrate secretion, thereby promoting Al tolerance in rice [36]. These findings suggest that GmMYB183 functions as a regulator of *GmMATE75* activity in Al tolerance. In addition, Arabidopsis MATE transporter 30 (AtDTX30) regulates auxin homeostasis in roots, influencing root development and Al tolerance [64]. The *dt*x*30* mutants show reduced elongation in primary roots, root hairs, and lateral roots, which is similar to *GmMYB183-OE* roots. The altered root architecture could be an adaptive response to metal stress, including Al stress, as root hairs can secrete citrate and create a rhizosphere environment that reduces Al toxicity. Moreover, citrate has been implicated in hormonal signaling pathways, particularly those related to auxin and ethylene, which play crucial roles in root development [65]. Therefore, we hypothesized that GmMYB183 modulates hormone levels in roots to regulate root development and promote Al tolerance.

The 14-3-3 proteins, discrete phosphateserine/threonine-binding modules, play crucial roles in regulating plant growth, development, and stress responses [66]. For instance, Ta*GRF6-A*, which encodes a 14-3-3 protein, enhances salt tolerance in wheat by interacting with TaMYB64 [67]. The interactions between the 14-3-3 protein and MYBS2 at specific serine residues play significant roles in regulating sugar-dependent nucleocytoplasmic shuttling and maintenance in the cytoplasm [68]. Moreover, inhibiting the interactions between 14-3-3 proteins and transcription factor PIF7 accelerates PIF7 dephosphorylation and promotes shade-induced nuclear localization, thereby enhancing the shade phenotype [69]. In this study, GmMYB183 in Arabidopsis roots was found to be located in the nucleus, cytoplasm, and cell membrane, while GmMYB183-S36A was only observed in the nucleus (Figure 3a). Additionally, unlike GmMYB183-S36A, GmMYB183 was found to bind to 14-3-3 protein (Figure 3b). These findings suggest that binding to 14-3-3 proteins alters the localization of GmMYB183. Similarly, the binding of soybean 14-3-3 proteins influences the intracellular localization of GmMYB176 and affects isoflavonoid synthesis [70]. However, the precise function of GmMYB183 after binding with 14-3-3 proteins remains undefined.

**Figure 9:**
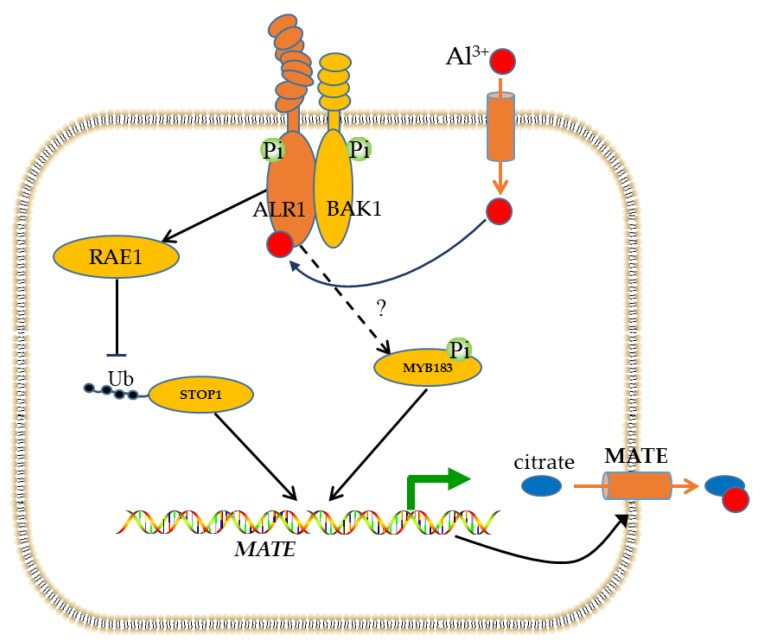
Schematic presentation of transcriptional regulation of *MATE* by GmMYB183 transcription factor. In response to Al stress, Al^3+^ interacts with a receptor (ALR1) on the plasma membrane to initiate a signaling pathway. Al signal phosphorylates GmMYB183 through an unknown pathway. Furthermore, the ALR1-BAK1 complex inhibits F-box protein (RAE1) activity, thereby preventing the degradation of STOP1. Both STOP1 and GmMYB183 activate the expression of the *MATE* gene, promoting the secretion of citrate and chelating Al ions.

The phosphorylation of transcription factors plays a crucial role in the regulation of gene expression and functions [71]. In plants, the ABA-dependent multisite phosphorylation of transcription activator AREB1 is essential for its own activity and the expression of ABA-inducible genes [72]. Freezing tolerance in Arabidopsis requires the MAPK6-mediated phosphorylation of the transcriptional repressor MYB15 [73]. Additionally, the phosphorylation of WRKY33 and ERF6 by MPK3/MPK6 activates defense-related genes and enhances the resistance against *Botrytis cinerea* [74,75]. Similarly, rhizobia inoculation in soybean inhibits the phosphorylation of GmMYB183 at Ser61 and enhances tolerance to salinity [76]. This study reveals that GmMYB183 regulates the expression of GmMATE75, potentially associated with the phosphorylation of GmMYB183 at Ser36, leading to the promotion of citrate secretion. Furthermore, the regulation of *GmMATE75* expression by GmMYB183 depends on two binding sites on the *GmMATE75* promoter (Figure 9). These findings highlight the involvement of GmMYB183 in response to different abiotic stresses through the phosphorylation of different sites. Therefore, evaluating the potential functions of GmMYB183-S36A under Al stress and identifying its upstream kinase of GmMYB183 and association with Al receptors are crucial, which provide valuable insights for the development of Al-tolerant crop varieties, contributing to sustainable agriculture and food security. 

## 5. Conclusions

In this study, we identified GmMYB183, a R2R3 MYB transcription factor in TBS, is involved in Al tolerance. Overexpression of *GmMYB183* in *Arabidopsis* and soybean hairy roots enhanced plant tolerance to Al stress compared to the wild type, with higher citrate secretion and less Al accumulation. Furthermore, using a dual-luciferase reporter system and yeast one hybrid, the GmMYB183 protein was shown to directly activate the transcription of *GmMATE75*. These results indicated that GmMYB183 is responsible for Al detoxification by promoting the secretion of citrate, providing valuable insight into the genetic basis for further elucidating the mechanism for improving plant tolerance to Al stress in acid soils. 

## Data Availability

Data are contained within the article or Supplementary Material.

## Supplementary Materials

The following supporting information can be downloaded at: https://www.mdpi.com/article/10.3390/biom14060724/s1, Figure S1: Citrate secretion from roots of TBS induced by Al stress. Figure S2: Transgenic Arabidopsis lines were screened. Figure S3: Bioinformatics for GmMYB183 transcription factor. Figure S4: Phylogenetic trees of GmMYB183 protein. Figure S5: Relative expression level of *GmMYB183* in Arabidopsis. Figure S6: Relative expression level of *AtMATE* in transgenic Arabidopsis. Figure S7: Different stages of the soybean hairy root transformation. Figure S8: Relative expression level of *GmMYB183* in hairy roots. Figure S9: Promoter sequence analysis of *GmMATE75*. Table S1: The primers used in this study. Table S2: The accession number of each protein in homologous alignment analysis.
