# Supplementary material for: GmMYB183, a R2R3-MYB Transcription Factor in Tamba Black Soybean (Glycine max. cv. Tamba), Conferred Aluminum Tolerance in Arabidopsis and Soybean
Source: Biomolecules. 2024 Jun 19;14(6):724. doi: 10.3390/biom14060724 (PMC11202213; doi:10.3390/biom14060724)
Supplement: Supplementary file 1 [file biomolecules-14-00724-s001.zip › biomolecules-3026583-supplementary/Supplementary figure.pdf]

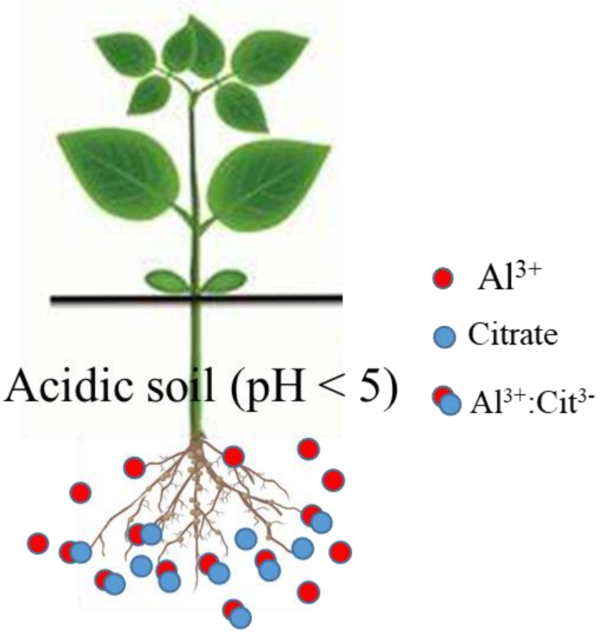

Figure S1. Citrate secretion from roots of TBS induced by Al stress. At pH < 5 in acid soil, Al can be released into the soil in the form of soluble Al^{3+} ion. Once TBS are subjected to Al stress, Al-induced root exudation of citrate into the rhizosphere, also forming Al-citrate complexes and thereby preventing Al^{3+} to enter into the roots.

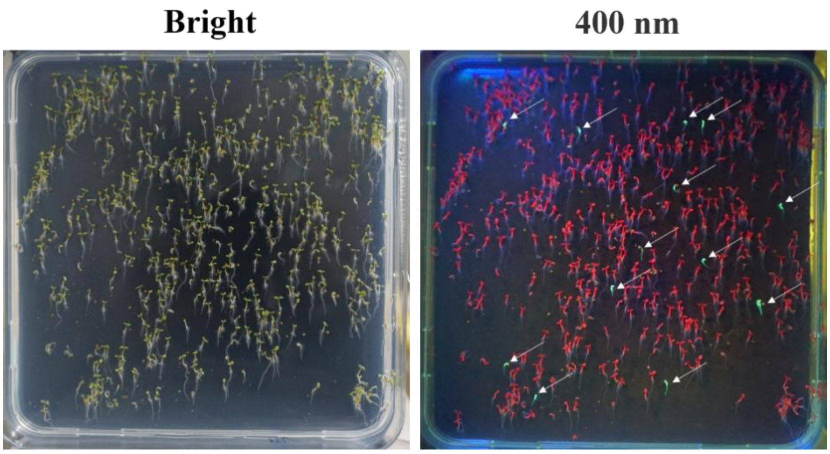

Figure S2. Arabidopsis seedlings of T0 generation with green fluorescence were screened out using LUYOR-3415RG hand-held fluorescent protein excitation light source (as indicated by the arrow).

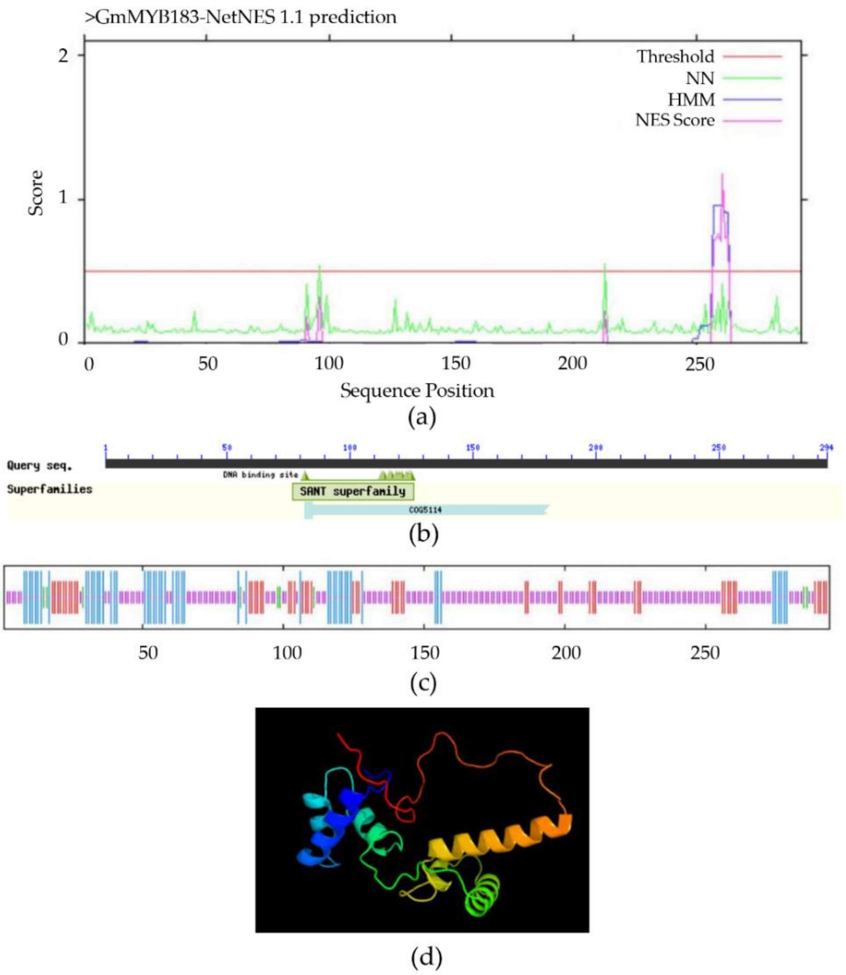

Figure S3. Bioinformatics for GmMYB183 transcription factor. (a) Nuclear localization signal prediction of GmMYB183. (b) Functional domain prediction of GmMYB183. (c) Secondary structure prediction of GmMYB183. (d) Predicted 3D structure of GmMYB183.

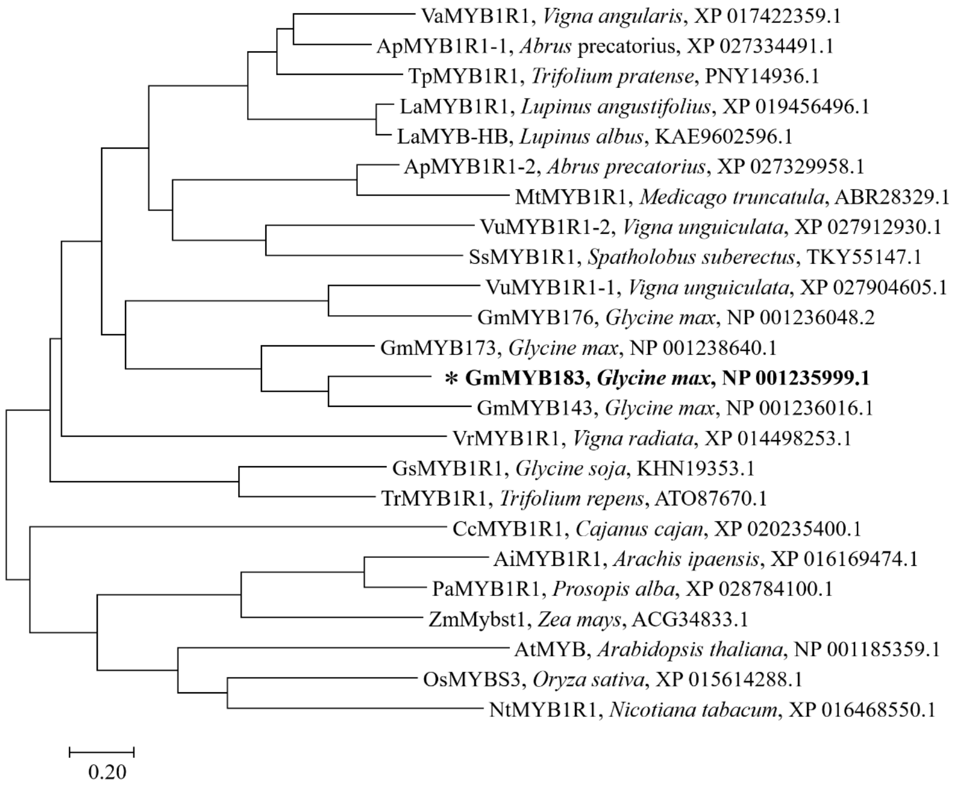

Figure S4. Phylogenetic trees of GmMYB183 protein. GmMYB183 is in bold with *.

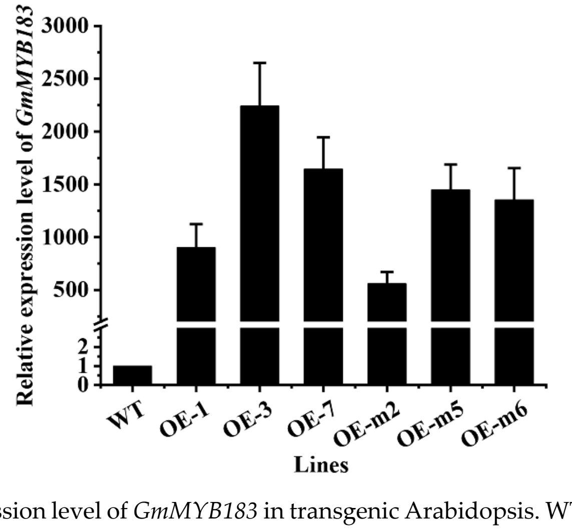

Figure S5. Relative expression level of *GmMYB183* in transgenic Arabidopsis. WT: wild type Arabidopsis. OE: Overexpression of *GmMYB183* in Arabidopsis, OE-m: Overexpression of *GmMYB183-S36A* in Arabidopsis.

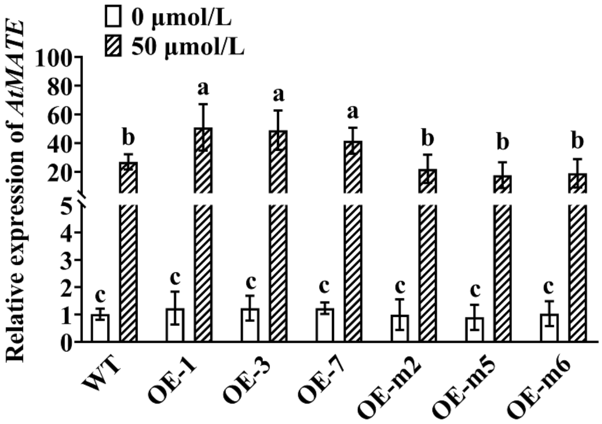

Figure S6. Relative expression level of *AtMATE* in transgenic Arabidopsis. WT: wild type Arabidopsis. OE: Overexpression of *GmMYB183* in Arabidopsis. OE-m: Overexpression of *GmMYB183-S36A* in Arabidopsis.

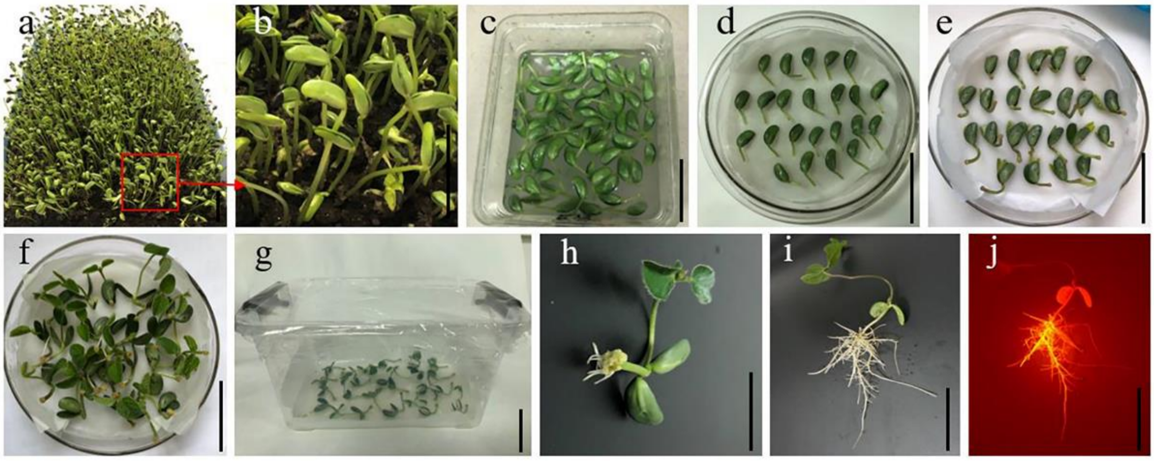

Figure S7. Different stages of the soybean hairy root transformation. (a) Germination for 4~5 day in soil. (b) The ideal stage for transformation: 5-day-old seedlings with unfolded cotyledons. (c) Inoculation with bacterial paste. (d) Moisturized cultivation. (e) Moisturized cultivation for 3 days. (f) Moisturized cultivation for 7 days. (g) Transferred to a large culture box for further moisturizing after 7 days. (h) Moisturized cultivation for 10-14 days. (i) Soybean plant 14 days after inoculation with sterile H_2O. (j) Screening of soybean transgenic hair roots. Scale bars in Figure S7 is 5 cm.

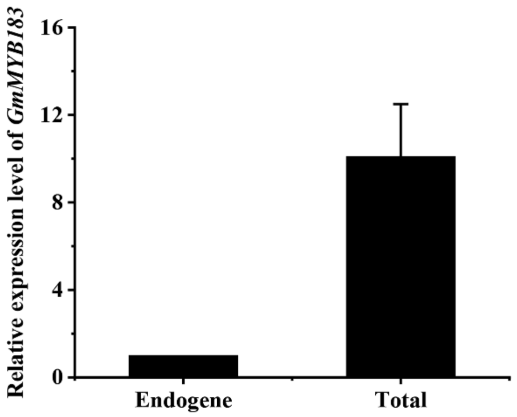

Figure S8. Relative expression level of *GmMYB183* in in hairy roots.

```
-1134  GTATATCAAC TAAAAGAGAG ACGATATCAT AATTTAGAGG GGGAGGGGGG AAATGCCCCT GTATCATATA
-1064  GGTATGGCTT GGTTGTGTAG AATATATGGA ATGAAAAAAA AAATTAACAA AAACATTTGA ATTAAAATAG
                  MYB
 -994  TGTAAAAAAG ATGTTTGATC TATATTAAAT AGTAAAAAAA ATTTGTCCAA CCCTTTCTCA TCAATTTCGT
 -924  CTCAAGCAAA CCAAGGGATA TATAACGTGA TCCCGTCCCT ATTTGTAGTT TGTACTATAT GCTCGATCGG
 -854  CTCTTACAAT TCTGTATATT TCAGTTCTAA CTGGGGAGCC ATTTGCATAT TTCTCGATGC ATGCATATAT
                                    Myb
 -784  AAAATAATAA TATACATTTT CGTTTGTTTC TACTTGATAG TTGTTACGAA GTTTCTATTA TGTTGCTATC
 -714  AAGGATATAT TTAAGGGACCTAA CTGAGGACGG TGCTTACTGT AGTGAGGATC TGTCACGATC TTCATTCAAC
                           Myb
 -644  TAACCCATCT TTGAATATTT AGATAGCATG CCATGGATCC TTCTCTAGCC TACATATTTT TTTATAGCAA
 -574  TTTAATGTCA TCAAGTGTTC ATTAGTA TACCAATTAA AAATAAAAAA TAGAAAACTT TTATTAAAAA
 -504  AATTATCAAT ACACCTAGCT GAAATTTCCG TAGCATCTCC AAATGTACTG ATTCCATAAT TAGTGTGTGA
 -434  TCACTGATTA TTAGCAAGAA ATTATTTTAA GTTATAAGGG TTTCTTTATA CTTAGAAGAT GAAATCTATA
 -364  GTTTAACTGA GGAAGGTTCC AACTATAAAA CAAAACAAAG TAATGATGAC TGTAACGTTT ACCAGGCTTA
         Myb
 -294  TTTAGTTGGC TCTTCGATAT CCCCAATGAT TCCTATAAAA ACCCATCACT TCTCTAGTGA TCTTTCAAAG
 -224  CCAAAAAAGG TACCACATTA TACTTTCTTA CACGTTTATT CTAGTTTGCC AACCTTGATT ACATGCAGTG
 -154  TGATCATATA TATATATATA TAAACAATAA TATTGGACAT ATTTAAACAT TGATATTGGA CGATCTCAAA
                             CAAT-box            TATA-box
  -84  CTCAAATTAT GATTAACTTG GCAGGTTGGT TGTTTCAAGC ACTTCAGTGT AGATCTCTCT CGCTTGTATG
          Transcription initiation site
  -14  AGATTCACTA TCCAATG······
                      +1
```

Figure S9. Promoter sequence analysis of *GmMATE75*. Red bold: Transcriptional initiation sites; Yellow background: TATA-box; Gray background: CAAT-box; Green background: MYB or Myb.
